# Ficoll density gradient sedimentation isolation of pelage hair follicle mesenchymal stem cells from adult mouse back skin: a novel method for hair follicle mesenchymal stem cells isolation

**DOI:** 10.1186/s13287-022-03051-3

**Published:** 2022-07-28

**Authors:** Yuyang Gan, Hailin Wang, Lijuan Du, Zhexiang Fan, Pingping Sun, Kaitao Li, Qian Qu, Jin Wang, Ruosi Chen, Zhiqi Hu, Yong Miao

**Affiliations:** Department of Plastic and Aesthetic Surgery, Nanfang Hospital of Southern Medical University, 1838 Guangzhou North Road, Guangzhou, Guangdong People’s Republic of China

**Keywords:** Hair follicle mesenchymal stem cells, Pelage hair follicle, Ficoll, Exosomes

## Abstract

**Background:**

Hair follicle mesenchymal stem cells (HF-MSCs) have great potential for cell therapy. Traditional method to isolate whisker HF-MSC is time-consuming and few in cell numbers. How to quickly and conveniently obtain a large number of HF-MSC for experimental research is a problem worth exploring.

**Methods:**

Two-step Ficoll Density Gradient Sedimentation (FDGS) was performed to isolate pelage HF-MSC from adult mice. The characteristic of the isolated cells was identified and compared with whisker HF-MSC by immunofluorescence staining, flow cytometry, three-lineage differentiation and hair follicle reconstruction. Pelage HF-MSC and exosomes were injected into the dorsal skin of mice as well as hair follicle organ culture to explore its role in promoting hair growth. The cells and exosomes distribution were located by immunofluorescence staining.

**Results:**

Isolated pelage HF-MSC expressed similar markers (ALP, Versican, NCAM, Nestin), showed similar growth pattern, possessed similar mesenchymal stem cells function and hair follicle induction ability as whisker HF-MSC. A large number of cells can be obtained with fewer mice compared to traditional method. Injected pelage HF-MSC promoted hair growth by secreting exosomes.

**Conclusion:**

A large number of Pelage HF-MSC can be isolated by FDGS, which can promote hair growth by secreting exosomes which may target the dermal papilla and hair matrix region of host hair follicle.

**Supplementary Information:**

The online version contains supplementary material available at 10.1186/s13287-022-03051-3.

## Introduction

Recent years, mesenchymal stem cells (MSCs) have been widely studied in tissue engineering and disease treatment [1–3]. The traditional sources of MSCs usually include bone marrow, fat, umbilical cord, etc. [4–8]. Finding new sources of mesenchymal stem cells has been a new research hot spot in recent years. Stem cells derived from hair follicle have attracted plenty of attentions [9, 10]. As the dermal component of hair follicle, dermal papilla (DP) is considered to be the niche of hair follicle mesenchymal stem cells (HF-MSC), which plays an important role in tissue regeneration and reprogramming in vitro [11–13]. At present, the HF-MSCs used for in vitro cell research and tissue engineering seed cells are derived from whisker hair follicles which using microdissection combined enzymatic digestion [14, 15]. However, as the difficulty of microdissection and small number of whisker hair follicle, the traditional isolation method of HF-MSC is time-consuming, difficult to operate, and the amount of cells is small. Dorsal skin contained a large number of hair follicle which could be the excellent donor region of HF-MSC [10]. However, due to the tiny size and massive number, traditional DPC isolation methods were not applicable, and the new method for isolation pelage HF-MSC has not been reported yet. Ficoll density gradient sedimentation (FDGS) is a classic method using to isolate different cell populations in peripheral blood, components were redistributed and aggregated in different layers of Ficoll according to the density gradient [16, 17]. Similarly, there are many mixed components in the back skin of mice. After enzyme digestion, the components with different densities are in the same suspension, using a specific Ficoll density gradient layer, pelage HF-MSC could be isolated.

In this study, we first proposed the method that used FDGS to isolate adult mice pelage HF-MSC, verified the purity of isolated stem cells and compared the similarities and differences in morphology, markers and functions between pelage and whisker HF-MSC. Further, we explored whether injected pelage HF-MSC and their exosomes could promote hair growth, and located the distribution of cells and exosomes in vivo, and revealed its possible mechanism.

## Materials and methods

### Animals

SPF 5-week-old C57BL /6 mice, SPF 5-week-old-male BALB/C mice and SPF C57BL /6 mice (1-3d) were purchased from the Experimental Animal Centre at Southern Medical University (Guangzhou, China).

### Isolation and culture of pelage DP cells

After removing the hair shaft on the back with shaver and depilatory paste, the mouse is carefully cleaned with 75% ethanol, the full layer of dorsal skin is cut off and panniculus carnosus is removed with toothed forceps. Then, the dorsal skin was rinsed with PBS containing 1% Antibiotic–Antimycotic (Gibco) three times and incubated with 0.2% collagenase I (Sigma-Aldrich) in TESCA buffer (Solarbio) for 1 h at 37 °C. The pelage DP were scraped off with micro-scissors and blown evenly in PBS, subsequently, centrifuging for 5 min at 1000 r/min, resuspending the pellet with 2 ml 5% Ficoll (Leagene) solution. Cautiously add, dropwise, 2 ml 5% Ficoll (Leagene) solution with pellet to a 15 ml centrifuge tube containing 2 ml 10% Ficoll (Leagene) solution. After density gradient sedimentation for 3 min at 200 r/min, collect the lower part and centrifuge for 5 min at 1000 r/min. Then, resuspend the pellet with DMEM (Gibco) and add slowly to a 15 ml centrifuge tube containing 2 ml 10% Ficoll (Leagene) solution. After density gradient sedimentation for 3 min at 150 r/min, collect the upper part and centrifuge for 5 min at 1000 r/min. Finally, resuspend the pellet with culture medium (DMEM with 20% FBS) and culture in the T25 flasks (Corning) at 37 °C 5%CO2 (Additional file 1: Fig. S1). All the pelage DPCs were cultured to passage 2(P2) for the further experiments.

### Isolation and culture of whisker DP cells

After cleaning with 75% ethanol, the whisker pads were cut off bilaterally with the scissor. Whisker pads were rinsed with PBS and then turned upside down and separated the follicle from the surrounding tissue using sharp forceps to expose the dermal side and hair follicle end bulbs. Then, a pair of blunt forceps is used to gently grip the most distal part of the hair follicle, before cutting away the end-bulb with micro-scissors and immediately placing in a sterile petri dish containing PBS. The dissected hair bulbs were rinsed with PBS and incubated with 0.2% collagenase I (Sigma-Aldrich) in TESCA buffer (Solarbio) for 1 h at 37 °C. After that, add a sufficient amount of culture medium, centrifuged (1000 r/min, 5 min, room temperature), discard supernatant and re-suspended with 20% culture medium, cultured in 6 cm culture dish. All the whisker DPCs were cultured to passage 2(P2) for the further experiments.

### Isolation of neonatal mouse epidermal cells

On postnatal day 1–3 (P1–3), mouse skin was cut off and cleaned by 75% ethanol. Then, it was rinsed with PBS containing 1% Antibiotic–Antimycotic for three times, and the skin was incubated with 0.1% dispase at 4 °C for 24 h. The epidermis was then peeled from the dermis and cut into pieces and incubated in 0.25% trypsin (Gibco) for 10 min at 37 °C to yield a single-cell suspension. Then, the single cell was re-suspended with PBS after centrifuging at 1000 r/min for 5 min prepared for hair follicle reconstruction.

### Observation on the morphology, adhesion and cell migration of dermal papilla

After observing the morphology of dermal papilla obtained from whisker and dorsal skin, DPCs were cultured for 7 days. The adhesion, migration, morphological characteristics and growth pattern of dermal papilla cells were observed.

### Immunofluorescence of specific markers

#### DP specific markers expression

DPCs pelage DP cells and single cell were fixed with 4% paraformaldehyde for 20 min, washed three times with TBST (Solarbio), and permeabilized with 0.1% Triton X-100 for 7 min at room temperature. After blocking with 5% BSA for 1 h, cells were incubated with a 1:200 dilution of Versican, Nestin, NCAM, ALP, Sox2 antibody (Bioss) overnight at 4 °C and subsequently incubated with a 1:200 dilution of 488-conjugated anti-rabbit IgG antibody (Abcam) for 1 h. Coverslips were mounted on glass slides using ProLong® Diamond Antifade Mountant with DAPI (Life Technologies, USA) prior to imaging with a confocal laser scanning microscope.

#### Sox2 expression of primary DP spheres

Whisker and pelage DP spheres were isolated according to the above methods. After isolation, DP spheres were fixed with 4% paraformaldehyde for 20 mins, washed and centrifugated with TBST three times, then permeabilized with 0.1% Triton X-100 for 30 min at room temperature. After blocking with 5% BSA for 1 h, 1:200 dilution of Sox2 antibody (Bioss) is carried out overnight at 4 °C and subsequently incubated with a 1:200 dilution of 488/594-conjugated anti-rabbit IgG antibody (Abcam) for 2 h, washed and centrifugated with TBST three times. DAPI was incubated for 30 min, washed and centrifugated with TBST three times, finally prepared to image with a confocal laser scanning microscope.

### Three-lineage differentiation of pelage and whisker DPCs

#### Adipogenic differentiation and oil red O staining

Briefly, DPCs were seed in 6-well plate at a density of 5 × 10^5/well, cultured in DMEM with 10% FBS. When the cell density in the well plate reached 100%, changed the culture medium to mouse mesenchymal stem cell adipogenic differentiation medium 1 (BG sciences, BGM-2533) (ADP1:Basal medium 175 ml, FBS 20 ml, Glutamin 2 ml, Pennicillin-Streptomycin 2 ml, Insulin 400 µl, IBMX 200 µl, Rosiglitazone 200 µl, Dexamethasone 200 µl) for 72 h, then changed the medium to ADP2 (ADP2: Basal medium 175 ml, FBS 20 ml, Glutamine 2 ml, Pennicillin-Streptomycin 2 ml, Insulin 400 µl) and cultured for 24 h. The two media were used alternately for 3–5 times, until enough lipid droplets appeared in the cells. Oil red staining was performed after differentiation. Plate was washed once with PBS, 4% paraformaldehyde fixed the cells for 30 min, then washed twice with PBS, incubated with Oil Red O staining solution at room temperature for 30 min. The cells were washed twice with PBS and then were imaged using inverted microscope.

#### Osteogenic differentiation and alizarin red staining

Briefly, DPCs were seed in 6-well plate at a density of 5 × 10^5/well, cultured in DMEM with 10% FBS for 24 h, changed the culture medium to mouse mesenchymal stem cell osteogenic differentiation medium (BG sciences, BGM-2522, include: basal medium 175 ml, FBS 20 ml, Glutamine 2 ml, Pennicillin-Streptomycin 2 ml, β-Glycerophosphate 2 ml, Ascorbate Acid 400 µl, Dexamethasone 20 µl). The medium was changed every 3 days. After 4 weeks induction, Alizarin red staining was performed. Plate was washed once with PBS, 4% paraformaldehyde fixed the cells for 30 min, washed twice with PBS, incubated with Alizarin red staining solution at room temperature for 30 min. The cells were washed twice with PBS and then were imaged using inverted microscope.

#### Chondrogenic differentiation and Alcian blue staining

Briefly, DPCs were seeded in 6-well plate at a density of 5 × 10^5/well, when cell density reached 90%, performed trypsin digestion, centrifugation, supernatant removal, added mouse mesenchymal stem cell chondrogenic differentiation medium (BG sciences, BGM-2544, including: basal medium 193 ml, Sodium pyruvate 200 µl, ITS supplement 2 ml, TGF-β_3_ 2 ml, Ascorbic acid 600 µl, Proline 200 µl, Dexamethasone 20 µl, Pennicillin-Streptomycin 2 ml), took half of the cell suspension (2.5 × 10^5 cells), centrifuged, loosened the centrifuge tube cap, cultured in incubator. Culture medium was changed every 3 days. After 4 weeks induction, Alcian blue staining was performed. Cell clusters were fixed with neutral formaldehyde, routinely paraffin-embedded sections. Paraffin section was dewaxed to water and rinsed with distilled water. Alcian blue staining solution was dyed for 30 min, then stopped staining with distilled water, imaged using inverted microscope.

### Hair follicle reconstitution assays

SPF 5-week-old male BALB/C mice were randomly divided into five groups (neonatal epidermal cells + pelage DPCs; neonatal epidermal cells + whisker DPCs; neonatal epidermal cells alone, pelage DPCs alone, whisker DPCs alone). The cells were re-suspended in 50 μL PBS. After successfully anesthetized, each point was injected 100 μL cell suspension (2.5 × 10^5 epidermal cells + 5 × 10^5 DPCs). Four weeks after injection, grafts were observed and taken for Paraffin sections.

### Isolation and identification of pelage HF-MSC and whisker HF-MSC exosomes

The culture medium supernatant of pelage HF-MSC and whisker HF-MSC were collected, 300 g centrifugation for 10 min, 2000 g centrifugation for 10 min to remove cells. Took the supernatant and pass through a 0.22 µM filter to remove cell fragments and vesicles with a diameter greater than 220 nm. Transfer the filtered supernatant to a new centrifuge tube at 4 °C to 100,000 g ultracentrifugation for 70 min. After centrifugation, remove the supernatant, used precooled 1 × PBS re-suspended at 4 °C and 100,000 g. Ultracentrifugation is done for 70 min. After centrifugation, exosomes were obtained. Using nanoparticle tracking analysis (NTA), the particle size of pelage HF-MSC exosomes was measured, and Nanosight NS300 software version 2.3 (Malven PANalytical) was used for analysis. The exosome was observed by Transmission Electron Microscope (TEM). Specific markers CD9 and CD81 were verified by western blotting.

### Injection of fluorescent labeled cells/exosomes and sections

#### Pelage and whisker HF-MSC injection

The pelage and whisker HF-MSC were labeled with DIO (Invitrogen) and injected into the dorsal skin of mice. After 7 days, the back skin of mice was taken and frozen section was performed and imaged with confocal laser scanning microscope.

#### Pelage and whisker HF-MSC exosomes injection

Exosomes of pelage HF-MSC and whisker exosomes were labeled with DIO (Invitrogen) and injected into the dorsal skin of mice every other day, three times injection in total, 1 × 10^10^ exosomes per injection. After 7 days, the back skin of mice was taken and frozen section was performed and imaged with confocal laser scanning microscope.

#### Hair follicle organ culture with exosomes

A complete mouse whisker hair follicles were isolated from the whisker pad. Exosomes were labeled by DIL (Invitrogen) and co-incubated with mouse whisker hair follicles. After 72 h, the hair follicles were sampled and frozen section was performed and imaged with confocal laser scanning microscope.

### Statistical analysis

All data were expressed as the means ± SD. Significance was tested with one-way ANOVA and homogeneity test of variance. *P* value < 0.05 was considered statistically significant.

## Results

### Pelage HF-MSC were isolated by FDGS

**Fig. 1:**
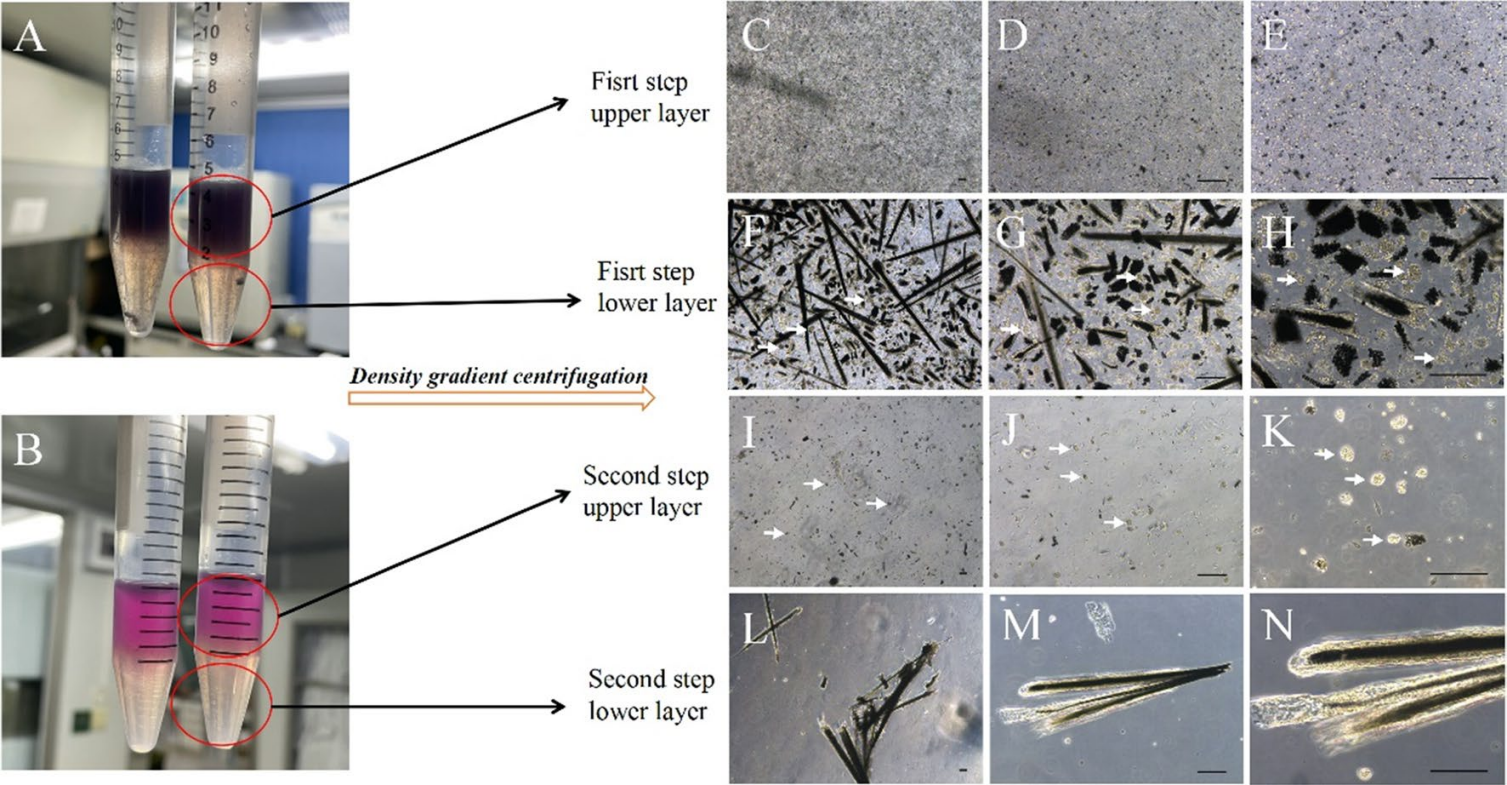
Ficoll density gradient sedimentation isolation of pelage DP sphere. After preparation of mouse back skin, digestion and scarping, FDGS was performed. In first step, 5% Ficoll mixed solution was added slowly on 10% Ficoll solution, which created two density layers (**A**). After first step centrifugation, upper layer presented a large number of single cells (**C**–**E**), while lower layer presented hair shafts and pelage DP spheres (**F**–**H**, white arrow). First step lower layer pellet was collected and re-suspended with 0% Ficoll solution (pure DMEM), slowly added on 10% Ficoll solution, which also created two density layers (**B**). After second step centrifugation, upper layer presented plenty of pelage DP spheres (**I**–**K**, white arrow), while lower layer presented unwanted hair shafts (**L**–**N**)

To isolate pelage DP spheres, we performed two-step FDGS isolation (Fig. 1, Additional file 1: Fig. S1). After preparation of back skin and removal penniculus carnosus, digestion and scarping, first mixed pellet was re-suspended with 5% Ficoll and added on 10% Ficoll to created first density layers (Fig. 1A). After first step gradient density sedimentation, unwanted single cells were removed in upper layer (Fig. 1C–E), pelage DP spheres and hair shafts were re-located at lower layer (Fig. 1F–H). Subsequently, collected first-step-lower layer pellet and re-suspended with DMEM (0% Ficoll), added on 10% Ficoll, create second density layers (Fig. 1B). Second step gradient density sedimentation isolated wanted pelage DP spheres in upper layer (Fig. 1I–K) and removed unwanted hair shafts in lower layer (Fig. 1L–N).

### Pelage HF-MSC showed typical emigration and growth pattern as whisker HF-MSC

To compare pelage and whisker, we first compared hair follicle structure. Compared with whisker pads (Additional file 1: Fig. S2A), dorsal back contained a large number of hair follicle with no blood sinus surrounded (Additional file 1: Fig. S2B). Second, we performed isolation of DP spheres. After enzyme digestion and scrapping, hair shaft, hair bulb and other impurities were mixed in the suspension (Additional file 1: Fig. S2C). After two-steps FDGS, abundant pelage DP spheres can be obtained (Additional file 1: Fig. S2D–F, red arrow). Whisker DP spheres were isolated by traditional microdissection and enzyme digestion, which showed unique structure different from pelage DP spheres (Additional file 1: Fig. S2G). Third, during primary cultured, Pelage DP sphere adhered and pelage HF-MSC started emigration in 24 h (Additional file 1: Fig. S2H) and almost finished emigration in 4 days (Additional file 1: Fig. S2I). Whisker HF-MSC started emigration in 4 days and almost finished in 7 days. Pelage DP sphere showed faster adhering and emigrating. Finally, during cultured, Pelage HF-MSC (Additional file 1: Fig. S2K) as well as whisker HF-MSC (Additional file 1: Fig. S2L) showed aggregation growth pattern. Above, pelage DP spheres we isolated showed typical emigration and aggregated growth pattern and possessed larger cell numbers and faster emigration time.

### Pelage HF-MSC expressed specific markers as whisker HF-MSC

**Fig. 2:**
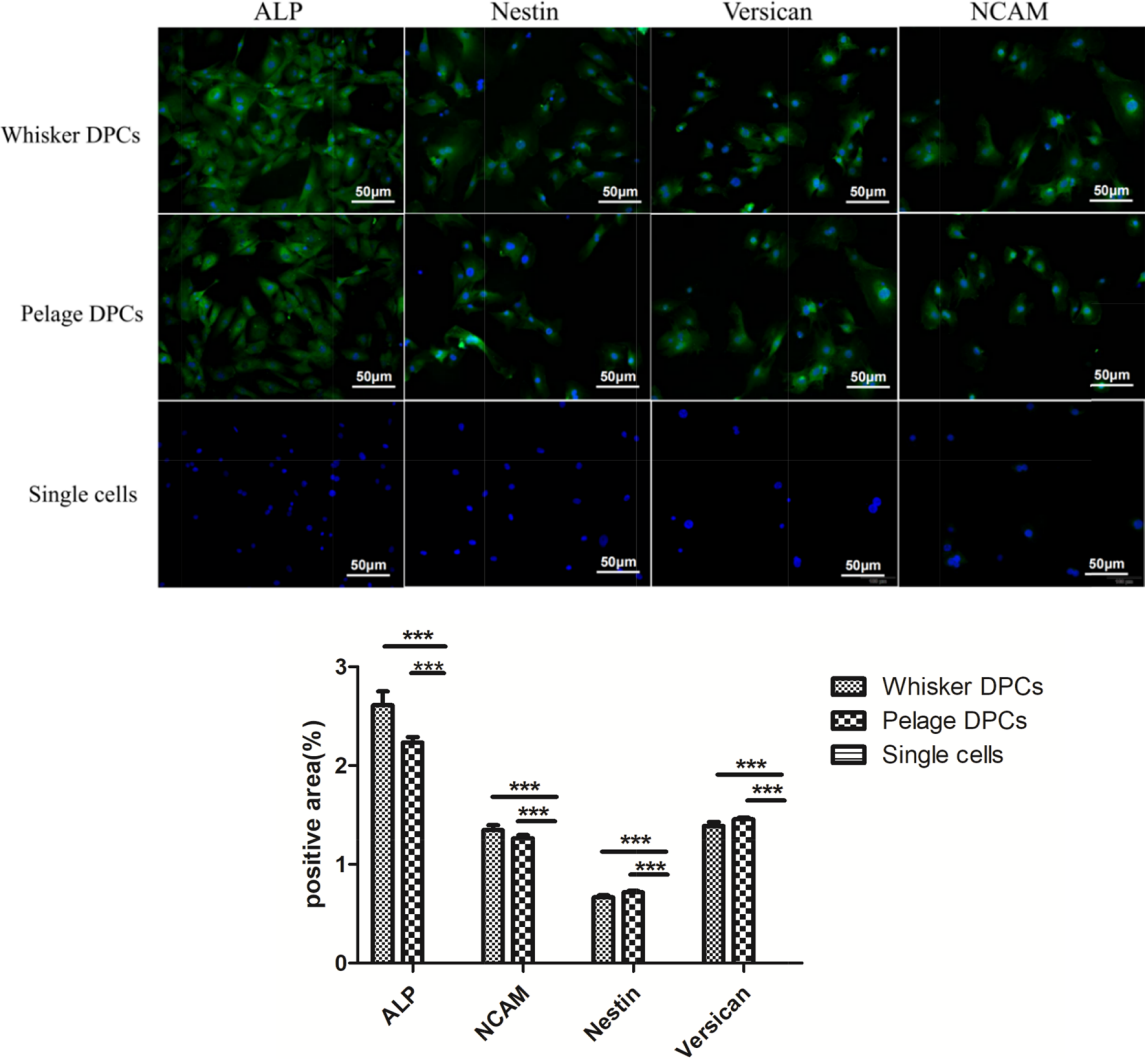
DP specific markers expression by Immunofluorescence. DP specific markers were verified by immunofluorescence. Whisker DPCs highly expressed ALP, Nestin, Versican and NCAM. Pelage DPCs also expressed those four markers and there was no significant difference in expression. Single cells that isolated in first step centrifugation didn’t express those markers

To verify the cells we isolated, we identified specific markers by immunofluorescence. After isolation and cultured, we collected upper layer single cells and pelage HF-MSC to verify markers, whisker HF-MSC worked as the control. ALP, Nestin, Versican and NCAM were believed to be the typical specific markers of HF-MSC in DP. As we can see in Fig. 2, pelage HF-MSC we extracted highly expressed those 4 markers, and there was no significant difference from whisker HF-MSC. Moreover, upper layer single cells we removed in first step didn’t express these markers. Above, Pelage HF-MSC showed similar high expression of specific markers as whisker HF-MSC and also further proved that what we isolated was pelage HF-MSC.

### Sox2 expression was different between Pelage and Whisker DP

To investigate Sox2 expression in both HF-MSC, we performed immunofluorescence of Sox2 in both primary DP sphere and cultured HF-MSC. After isolation, primary DP spheres were immediately performed staining without culture. Whisker DP sphere showed high Sox2 expression (Additional file 1: Fig. S3A–C), while Pelage DP sphere showed almost no expression of Sox2 (Additional file 1: Fig. S3D–F). Further, we cultured both HF-MSC to detected Sox2 expression again. Consistently, cultured whisker HF-MSC still highly expressed Sox2 (Additional file 1: Fig. S3G–I), while over 80% pelage HF-MSC showed almost no expression of Sox2 (Additional file 1: Fig. S3J–L). Above, there was significant difference between pelage DP and whisker DP in the expression of Sox2.

### Pelage HF-MSC expressed the same MSCs specific markers and possessed the same three-lineage differentiation ability as whisker HF-MSC

**Fig. 3:**
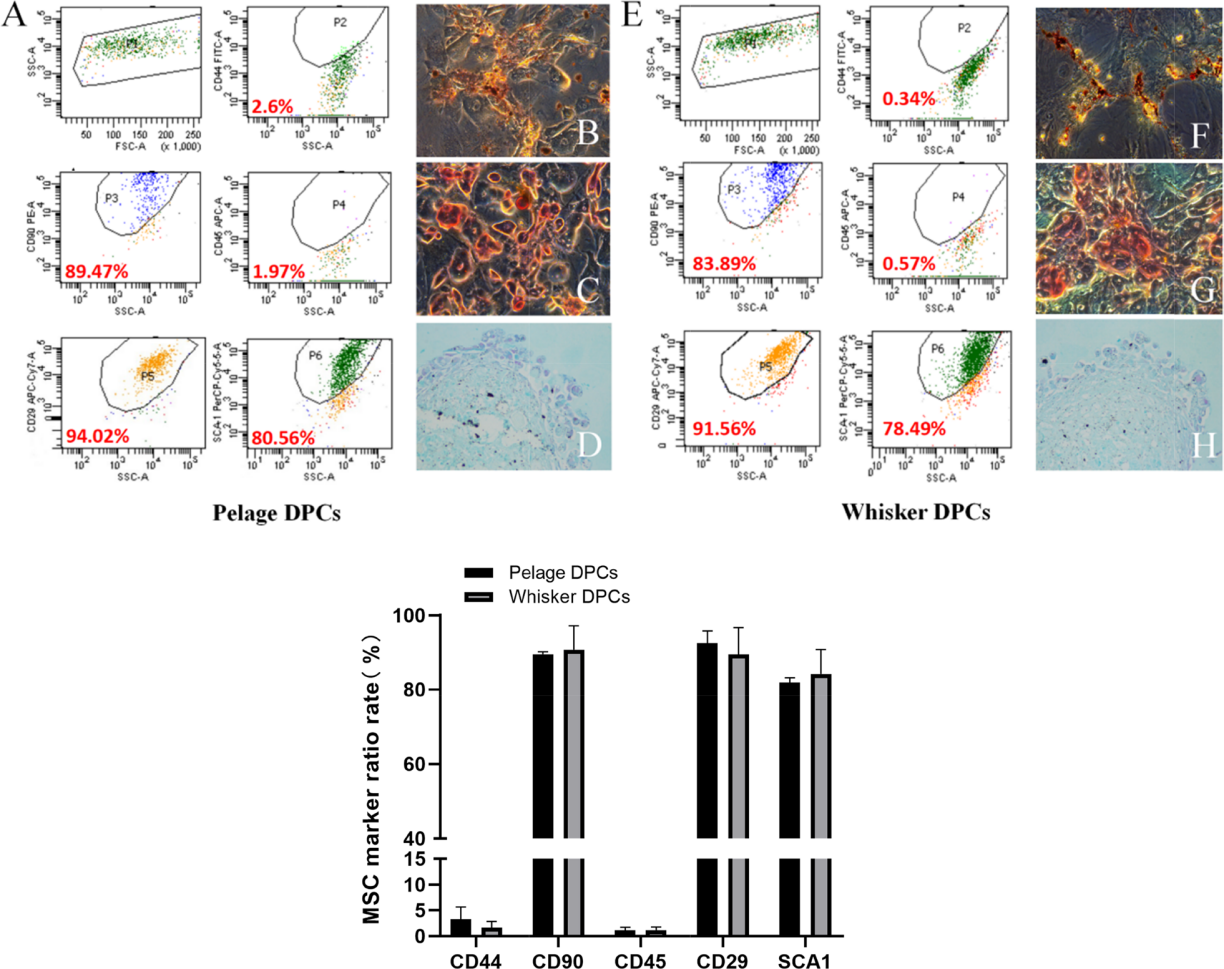
MSC Characteristics of Pelage DPCs and Whisker DPCs. Flow cytometry tested for mouse MSC specific markers and three-linage differentiation was performed. Pelage DPCs highly expressed CD29 (94.02%), CD90 (89.47%) and Sca-1 (80.56%), but hardly expressed CD45 (1.97%) and CD44 (2.6%) (**A**). Pelage DPCs showed adipogenic (**B**, Oil Red O staining), osteogenic (**C**, Alizarin Red staining) and chondrogenic (**D**, Alcian Blue staining) differentiation ability. As the control, whisker DPCs showed similar MSC markers expression (**E**) and also similar ability of adipogenic (**F**), osteogenic (**G**) and chondrogenic (**H**) differentiation

To verify the MSCs characteristic of isolated pelage HF-MSC, specific CD markers and three-lineage differentiation ability were identified. After isolation and culture, MSC specific cell surface markers were identified by flow cytometry. Pelage HF-MSC highly expressed CD90, CD29 and Sca-1, while hardly expressed CD45 and CD44 (Fig. 3A), the results were consistent with whisker HF-MSC (Fig. 3E). Moreover, three-lineage differentiation ability was believed to be the most important verification of MSC characteristic. Pelage HF-MSCs were cultured in adipogenic differentiation medium for 1 week that lipid droplets can be found in the cells, oil red o staining verified adipose formation (Fig. 3B, F). As for osteogenic and chondrogenic differentiation, Pelage HF-MSCs were cultured in osteogenic or chondrogenic differentiation medium for 4 weeks. Alizarin red staining verified bone nodule formation (Fig. 3C, G). Alcian blue staining verified successful cartilage differentiation (Fig. 3D, H). Meantime, whisker HF-MSC also presented similar three-lineage differentiation ability which has no significant difference from pelage HF-MSC. Above, we confirmed pelage HF-MSC expressed MSC specific cell surface markers and possessed similar three-lineage differentiation ability as whisker HF-MSC.

### *Pelage HF-MSC had the ability to induce hair follicle reconstruction *in vivo

**Fig. 4:**
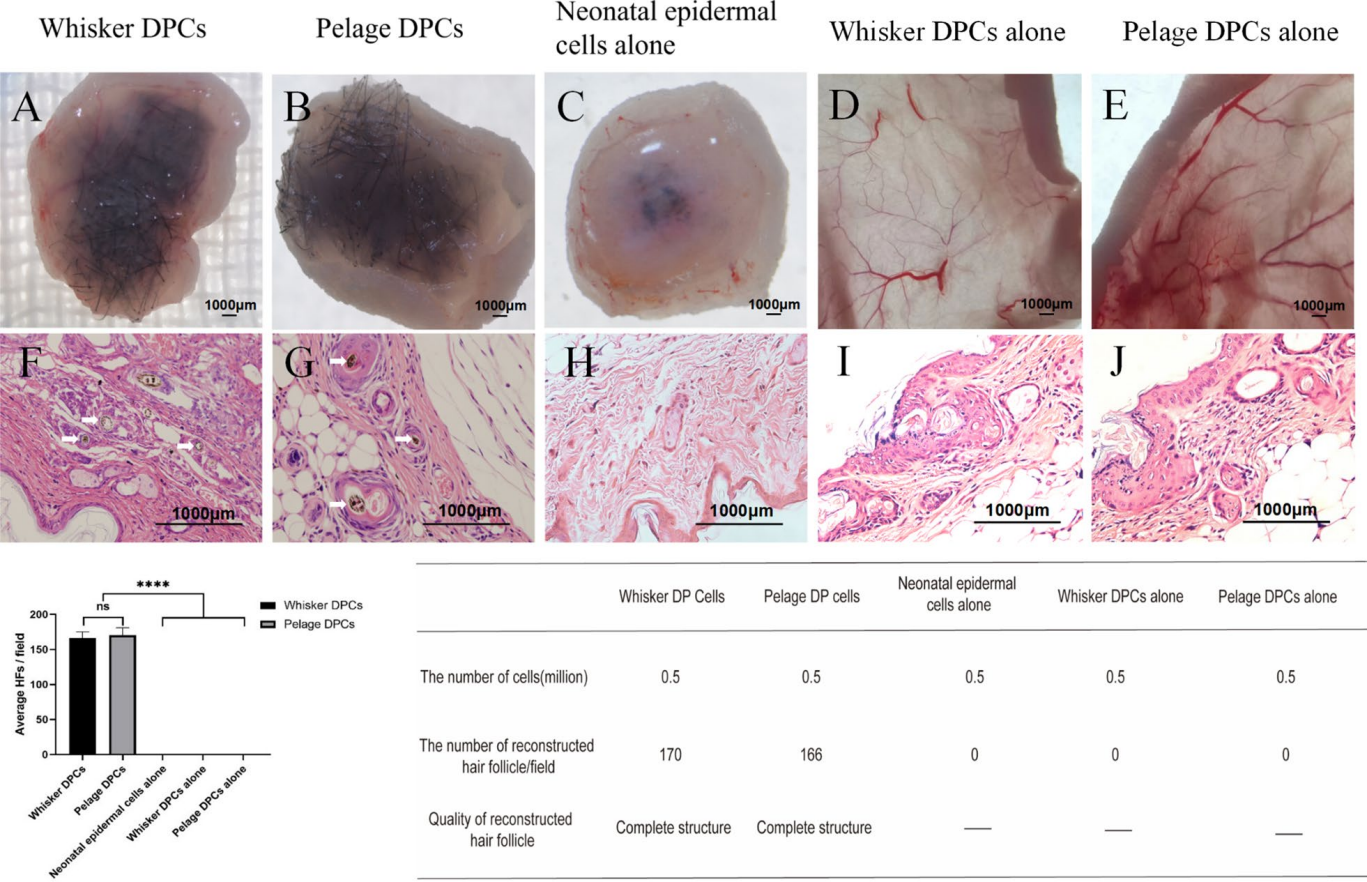
Hair follicle reconstruction ability of Pelage DPCs and Whisker DPCs. DPCs were fixed with neonatal mice epidermal cells and were injected into nude mice to test hair follicle reconstruction ability. Complete hair follicle can be seen after 4 weeks injection by Whisker DPCs induced (**A**, **F**, white arrow indicated reconstructed hair follicle). Pelage DPCs can also reconstruct complete structure hair follicle as the Whisker DPCs did (**B**, **G**, white arrow indicated reconstructed hair follicle). As the control, neonatal epidermal cells alone failed to reconstruct hair follicle (**C**, **H**), and also, both DPCs alone failed to reconstruct hair follicle (**D**, **E**, **I**, **J**). Average HFs of Whisker and Pelage DPCs induced hair follicle reconstruction showed no significant difference under the same amount of injected cells. Both DPCs possessed the ability to reconstruct hair follicle with complete structure

To verify inducing ability in vivo, Pelage HF-MSC and whisker HF-MSC were used to test the ability of hair follicle reconstruction. 4 weeks after injection, both pelage HF-MSC + epidermal cells group and whisker HF-MSC + epidermal cells group formed complete hair follicle structure (Fig. 4A, B), while epidermal cells alone and both DPCs alone failed to reconstruct hair follicle as control (Fig. 4C–E). HE staining also confirmed complete hair follicle structure by both HF-MSC inducing (Fig. 4F, G, white arrow). Under the same cells numbers, average number of reconstructed hair follicles and structure showed no difference. Above, pelage HF-MSC showed in vivo hair follicle reconstruction inducing ability.

### Pelage HF-MSC injection promote hair growth and located around the host hair follicle

**Fig. 5:**
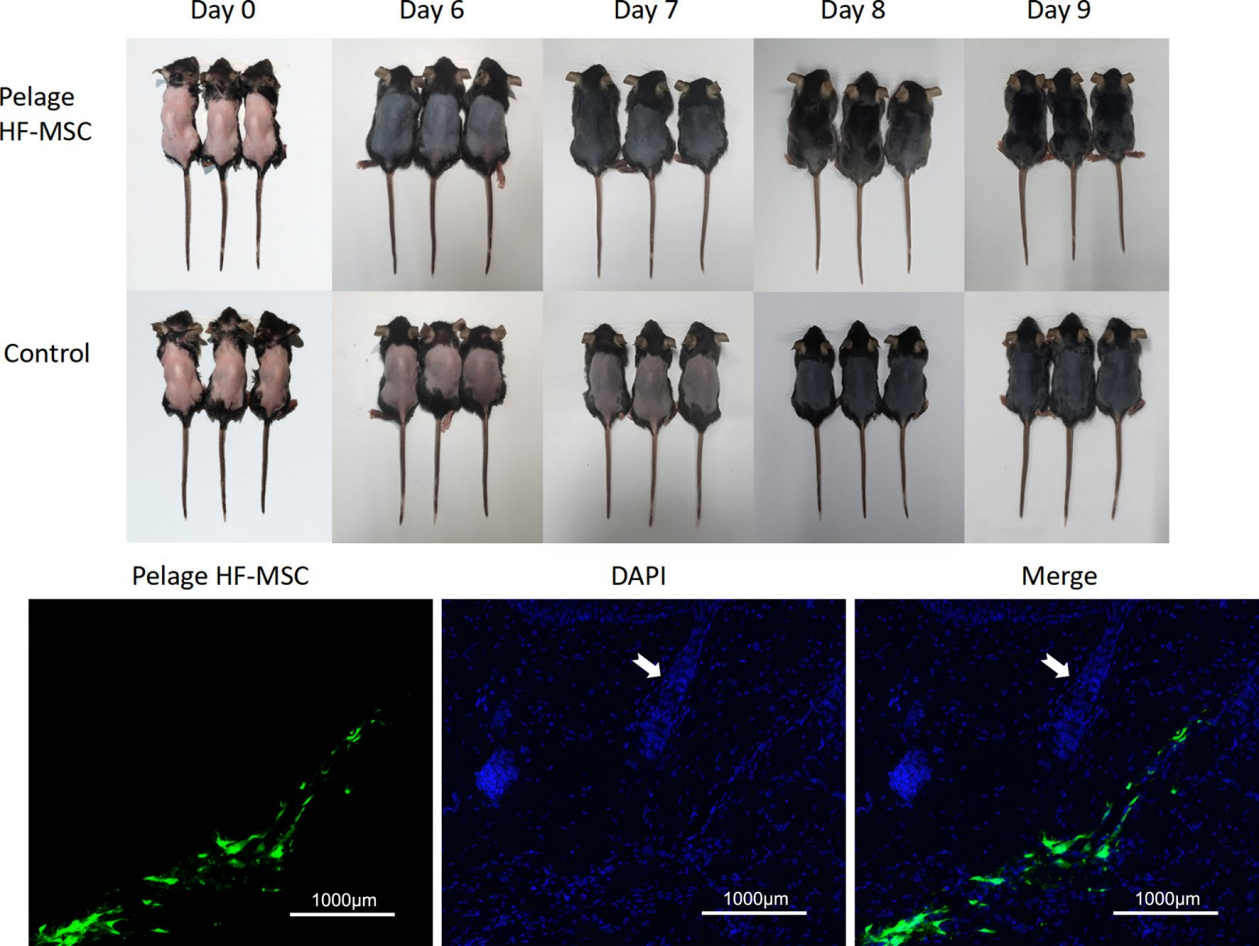
Effect and distribution of pelage HF-MSC treatment on dorsal hair growth. 3-week-old C57BL/6 mice were divided into Pelage HF-MSC group and control group. 6 days after intradermal injection, Pelage HF-MSC group enters into anagen period earlier than control group. Labeling pelage HF-MSC with DIO (green) showed that pelage HF-MSC located around the host hair follicle (White arrow)

In order to explore whether Pelage HF-MSC could be used as seed cells of cell therapy to promote hair growth, we injected pelage HF-MSC into the dorsal skin of mice. 6 days after intradermal injection of pelage HF-MSC, experiment group entered the anagen earlier than the control group (Fig. 5), while whisker HF-MSC showed the same ability to promote hair growth (Additional file 1: Fig. S5). In order to further explore the distribution of pelage HF-MSC after injection, we labeled HF-MSC with DIO. As shown in Fig. 5, most of pelage HF-MSC located around the host hair follicle, and few of them integrated into the host hair follicle, whisker HF-MSC showed the similar in vivo localization (Additional file 1: Fig. S5). Above, it is suggested that pelage HF-MSC’s role in promoting hair growth may be related to the paracrine effect of the cells distributed around the host hair follicle.

### *Pelage HF-MSC exosomes promote hair growth *in vivo* and *in vitro

**Fig. 6:**
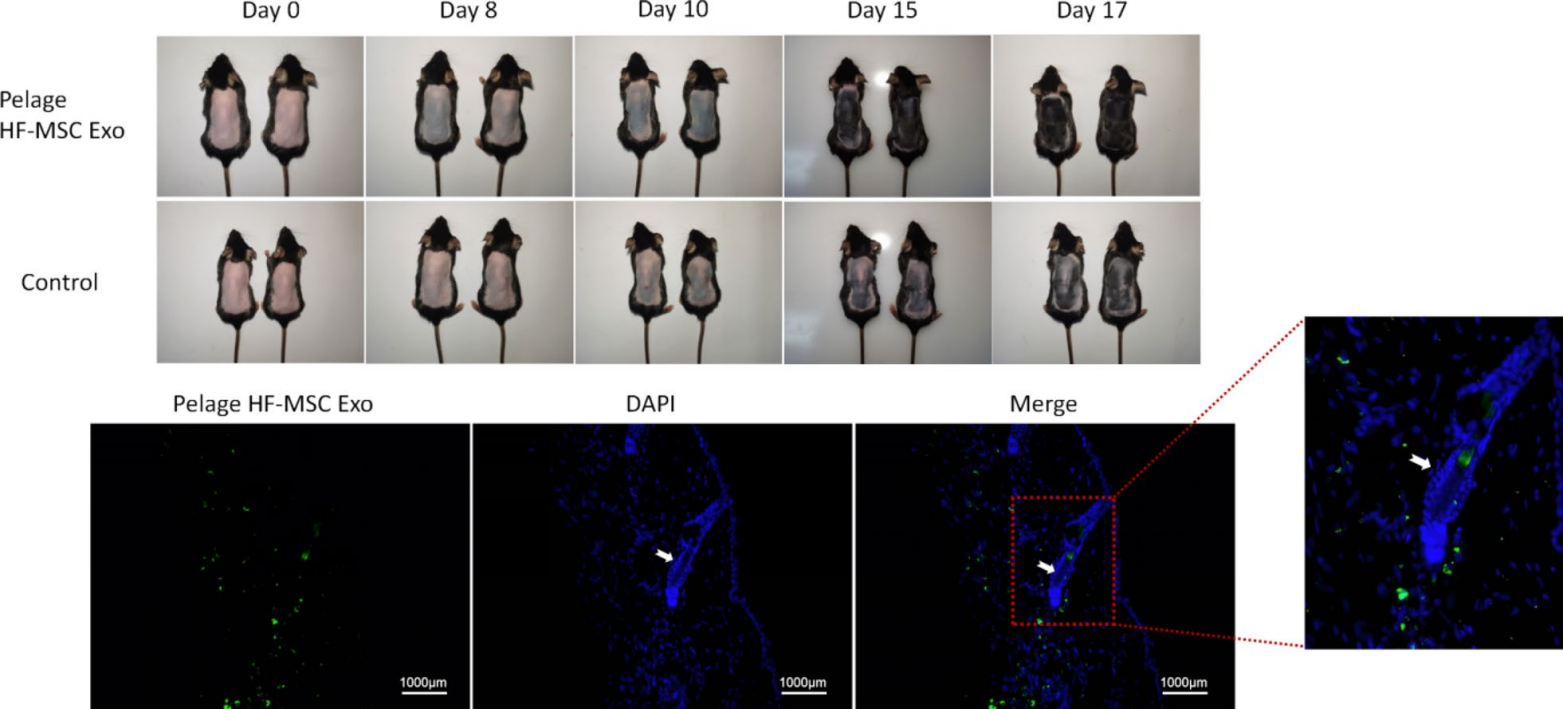
Effect and distribution of pelage HF-MSC Exosomes treatment on dorsal hair growth. 7-week-old C57BL/6 mice were divided into Pelage HF-MSC exosomes group and control group. Exosomes were injected every other day, a total of 3 times injection were performed. On day 8, pelage HF-MSC exosomes group enter into anagen period earlier than control group. Injected pelage HF-MSC exosomes were labeled with DIO (green) and located around host hair follicle (white arrow)

In order to further explore the paracrine effect of pelage HF-MSC, based on previous literature, we isolated the exosomes of pelage HF-MSC. The isolated exosomes were observed by electron microscope, the expressions of related markers CD81 and CD9 were verified by WB. The particle size was between 30 and 100 nm (Additional file 1: Fig. S4). In order to further explore the effect of exosomes on hair growth, we first injected exosomes into the dorsal skin of mice. As shown in Fig. 6, after exosomes injection, pelage HF-MSC exosomes treatment group entered the anagen earlier than the control group, while whisker HF-MSC exosomes group showed similar effect (Additional file 1: Fig. S6). Further in vivo fluorescence staining showed the localization of the injected exosomes in the skin (Fig. 6 for pelage HF-MSC exosomes, Additional file 1: Fig. S6 for whisker HF-MSC exosomes). Above, it is suggested that pelage HF-MSC secreted exosomes to promote hair growth in vivo.

**Fig. 7:**
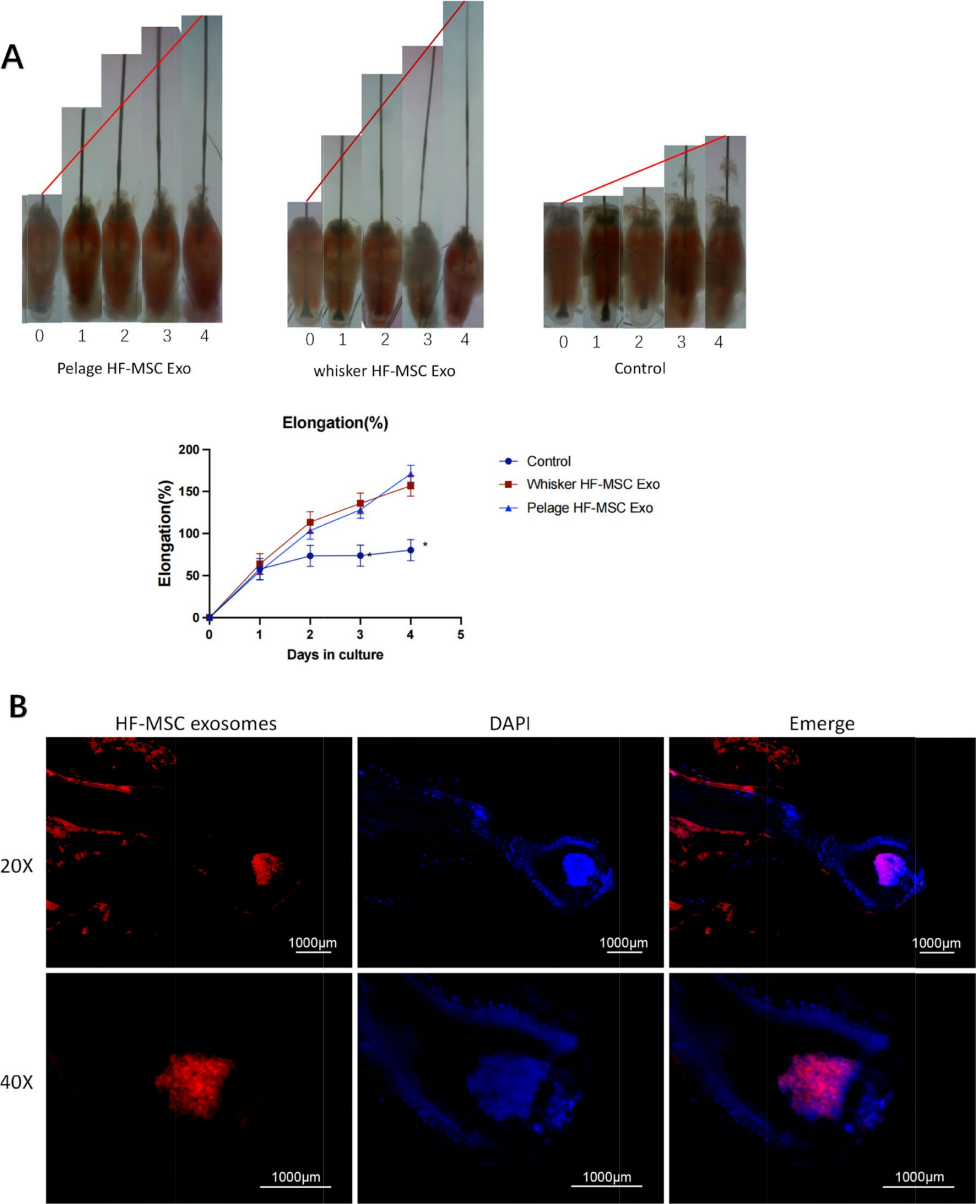
Exosomes of pelage HF-MSC promoted hair growth and integrated into host hair follicles. The exosomes of pelage HF-MSC were labeled with DIL (Red) and co-cultured with mouse hair follicles. Exosomes can promote the growth of mouse hair follicle (**A**). And, after 72 h, the exosomes (Red) of pelage HF-MSC were ingested by hair follicles and located at the dermal papilla and hair matrix region

Then, we co-cultured the exosomes and mouse hair follicle in vitro organ culture. As shown in Fig. 7A, pelage HF-MSC exosomes promote hair growth in vitro compared to control group. And also, whisker HF-MSC exosomes showed the same hair growth promoting ability. In order to further explore the effect of exosomes acting on hair follicles, we labeled pelage HF-MSC exosomes with DIL. After 72 h co-cultured, it can be seen that pelage HF-MSC exosomes integrated into hair follicles and specifically integrated into dermal papilla and hair matrix region (Fig. 7B), suggesting that exogenous HF-MSC may secrete exosomes and target at the hair matrix and dermal papillae region of the host hair follicle to promote hair growth.

## Discussion

Dermal papilla cells (DPCs) worked as hair follicle mesenchymal stem cells (HF-MSC) that are believed to be the important seed cell for hair follicle regeneration, tissue engineering and stem cell therapy [18, 19]. Because it has the characteristics of MSCs and the ability to be reprogrammed [20–25], massive number of DPCs were needed for research.

As for mouse dorsal skin, enzyme digestion could lead to several components such as single cells, collagen tissue and different hair follicle elements. According to the isolation method we proposed, pelage DP sphere needs to be separated from single cells (fibroblast mainly) and hair shaft. Density gradient of these three components from large to small was hair shaft, DP sphere, single cells. In order to obtain purified DP, we set two-steps isolated procedure. Based on the our previous research (unpublished data), 0%, 5% and 10% Ficoll solution concentration gradient were set. First step was used to remove single cells, low density lead single cells stay at 5% Ficoll layer, while DP sphere and hair shaft sank to 10% Ficoll layer. Second step was used to remove hair shaft, high density lead hair shaft sank to 10% Ficoll layer, while DP sphere stayed at 0% Ficoll layer.

Once the isolation step finished, pelage DP spheres could be observed (Fig. 1). Further, the emigration and aggregation growth pattern, as well as specific markers expression, verified the part we isolated was pelage DPCs (Additional file 1: Fig. S2). Moreover, MSCs characteristics of pelage DPC were also verified. Pelage DPC expressed MSCs specific CD markers (Fig. 2), which was almost consistent of whisker DPCs. Further, basic MSCs three-lineage differentiation potential were studied that pelage DPCs can differentiate into adipocytes, osteocyte, chondrocytes. Markers expression and differentiation verification confirmed that pelage DPC was one subset of hair follicle mesenchymal stem cells (HF-MSCs) [26, 27]. Finally, hair follicle inducing ability of pelage DPC, which was one of the most important function of DPCs, was also verified. By mixing pelage DPCs and epidermal cells, complete hair follicle could be reconstructed in vivo (Fig. 4). We proposed a novel method to isolated pelage DPC, and DP markers, MSCs characteristics, inducing function was performed to verify the accuracy of DPCs.

Compared with whisker DPCs, both DPCs expressed traditional DP markers such as ALP, versican, ncam, nestin [28–31]. Interestingly, pelage DP possessed obvious different expression on Sox2. As previously literature reported, Sox2 determine different hair types in mice [19]. Pelage hair follicle presented over 90% zigzag hair follicle which determined to be Sox^2−^ dermal papillae type, while whisker hair follicle owned Sox^2+^ dermal papilla that restricted to guard, awl and auchene hair type [32–34]. Our results were consistent with previous reported, the pelage DPCs we isolated showed almost no Sox^2^ expression. In addition, absence of Sox^2^ did not affect hair follicle formation. Our pelage DPCs showed similar ability of in vivo hair follicle reconstruction as whisker Sox^2+^ DPCs, as hair follicle reconstruction ability was one of the most important index of DPCs function. Meanwhile, MSC characteristic was also not affected by Sox^2^. Pelage DPCs owned three-lineage differentiation ability, which indicated that So^x^2 in hair follicle dermal papilla may no longer be an absolute sign of maintaining stemness, instead, it controlled hair growth and development pattern, decided the specific hair type [35–38].

Significantly, pelage DP started emigration in 24 h while whisker DP started at day 3. This may be due to the smaller size and unique structure of pelage DP. Especially important, traditional whisker DPC primary culture usually needs 5 to 10 C57BL/6 mice for one 6 cm petri dish, cost 10 to 14 days for emigration and proliferating to 5 × 10^5^ cells. The whole isolation procedure cost about 4 h. But as for pelage DP isolated procedure we proposed, only one C57BL/6 mouse can provide two T25 culture flasks DPCs, just need 4–7 days for emigration and proliferating to 1 × 10^6^ cells. The whole isolation procedure only cost about 2 h.

Further, we explored whether pelage HF-MSC possessed the same ability of promoting hair growth as the whisker HF-MSC. By labeling the injected cells for in vivo localization, it showed that the cells were distributed around the host hair follicle, which may promote hair growth by paracrine effect. Further labeling and injection of exosomes showed that exosomes promoted hair growth in vivo and in vitro. This was consistent with the literature reported by Hu et al. in 2020 [18], that exosomes secreted by whisker HF-MSC were important factors to promote hair growth. Interestingly, during in vivo experiments, part of the exosomes injected into the mouse dorsal skin were located around the host hair follicle, while the other part was integrated into host hair follicle. However, during in vitro organ culture experiments, exosomes co-cultured with mouse hair follicles could be ingested by hair follicles in large quantities and located specifically in the dermal papilla and hair matrix region. This difference in distribution in vivo and in vitro may be due to the fact that dermal papilla cells themselves were a special kind of fibroblasts, the exosomes derived from DPC may be internalized and ingested by fibroblasts around the hair follicle. However, under the condition of hair follicle organ culture in vitro, without fibroblasts around the hair follicle and also the dermal sheath gradually disintegrated during culture, the co-cultured exosomes can be ingested by the hair follicles and targeted at the dermal papilla and the hair matrix region. In the future, how to better deliver exogenous exosomes around hair follicles in vivo is worth exploring.

Above all, we provided a convenient method to obtain a large number of pelage DPCs, because there was no significant difference in MSCs characteristics, inducing ability and hair promotion ability between the two DPCs and also pelage DPCs could promote hair growth through exosomes secreting, pelage DPCs can provide large numbers of HF-MSCs for further research such as stem cell therapy, tissue engineering and 3D print seed cells. This time-saving and efficient isolation method may provide great convenience for follow-up research.

## Conclusion

FDGS combined enzyme digestion is an efficient and time-saving method for adult mice pelage DPCs isolation, which provided a great support to the research of hair follicle mesenchymal stem cells.

## Supplementary Information

**Additional file 1: Fig. S1**. Schematic diagram of Pelage DPCs isolation. **Fig. S2**. Morphology, adhesion and cell migration of dermal papilla cells. Whisker (A) and Pelage (B) follicles morphology observed under stereoscope, no blood sinus was found in pelage follicle, the number of pelage follicles was much more than whisker follicles. Hair shafts and hair bulb located in the same suspensions after enzyme digestion and scarping (C). After two-steps isolation, several Pelage DP spheres can be seen under microscope (D-F, Red arrow). Whisker DP spheres were observed under microscope (G, white arrow). Pelage DPCs started emigration in 24 h (H, white arrow showed DPCs started emigrated out of the DP sphere) and proliferation, most of pelage DPCs finished emigration in 4 days (I, white arrow showed plenty of DPCs emigrated out of the DP sphere), while whisker DPCs started emigrate in 72 h and finished in 7 days (J). Pelage DPCs (K) and whisker DPCs (L) showed similar cell morphology and aggregation growth pattern. **Fig. S3**. Differential expression of Sox2 between whisker and pelage DPCs. Sox2 expression was detected in primary DP sphere and cultured DPCs. Whisker DP showed high Sox2 expression both in DP sphere and DPCs, while Pelage DP showed almost no expression on Sox2 whether in primary DP sphere or cultured DPCs. **Fig. S4**. Isolation and identification of Pelage HF-MSC and whisker HF-MSC exosomes. The morphology of exosomes was observed by electron microscope; Western Blotting verification specific markers CD9 and CD81; The detection particle size was between 30 and 100 nm. **Fig. S5**. Effect and distribution of Whisker HF-MSC treatment on dorsal hair growth. 3-week-old C57BL/6 mice were divided into Pelage HF-MSC group and control group. Pelage HF-MSC group enter into anagen period earlier than control group, which was similar to Pelage HF-MSC group. Labeling whisker HF-MSC with DIO (green), showed that whisker HF-MSC located around the host hair follicle (White arrow). **Fig. S6**. Effect and distribution of whisker HF-MSC Exosomes treatment on dorsal hair growth. 7-week-old C57BL/6 mice were divided into whisker HF-MSC exosomes group and control group (The same as the control group in Fig. 6). Exosomes were injected every other day, a total of three times injection were performed. On day 8, whisker HF-MSC exosomes group enter into anagen period earlier than control group. Injected whisker HF-MSC exosomes were labeled with DIO (green) and located around host hair follicle (white arrow). **Fig. S7**. Pictorial diagram to show the location of MSCs and exosomes injection protocol. The cell/exosomes suspension was injected along the midline of the back of the mouse, and the amount of cell suspension was 300 µl. HF-MSCs suspension injection was performed once, exosomes suspension injection was performed.

## Data Availability

The data used to support the findings of this study are available from the corresponding author upon request.

## Abbreviations

FDGS: Ficoll density gradient sedimentation
HF-MSC: Hair follicle mesenchymal stem cells
DPC: Dermal papilla cells
