# Supplementary material for: Ficoll density gradient sedimentation isolation of pelage hair follicle mesenchymal stem cells from adult mouse back skin: a novel method for hair follicle mesenchymal stem cells isolation
Source: Stem Cell Res Ther. 2022 Jul 28;13:372. doi: 10.1186/s13287-022-03051-3 (PMC9330686; doi:10.1186/s13287-022-03051-3)

**Figure S1 Schematic diagram of Pelage DPCs isolation.**


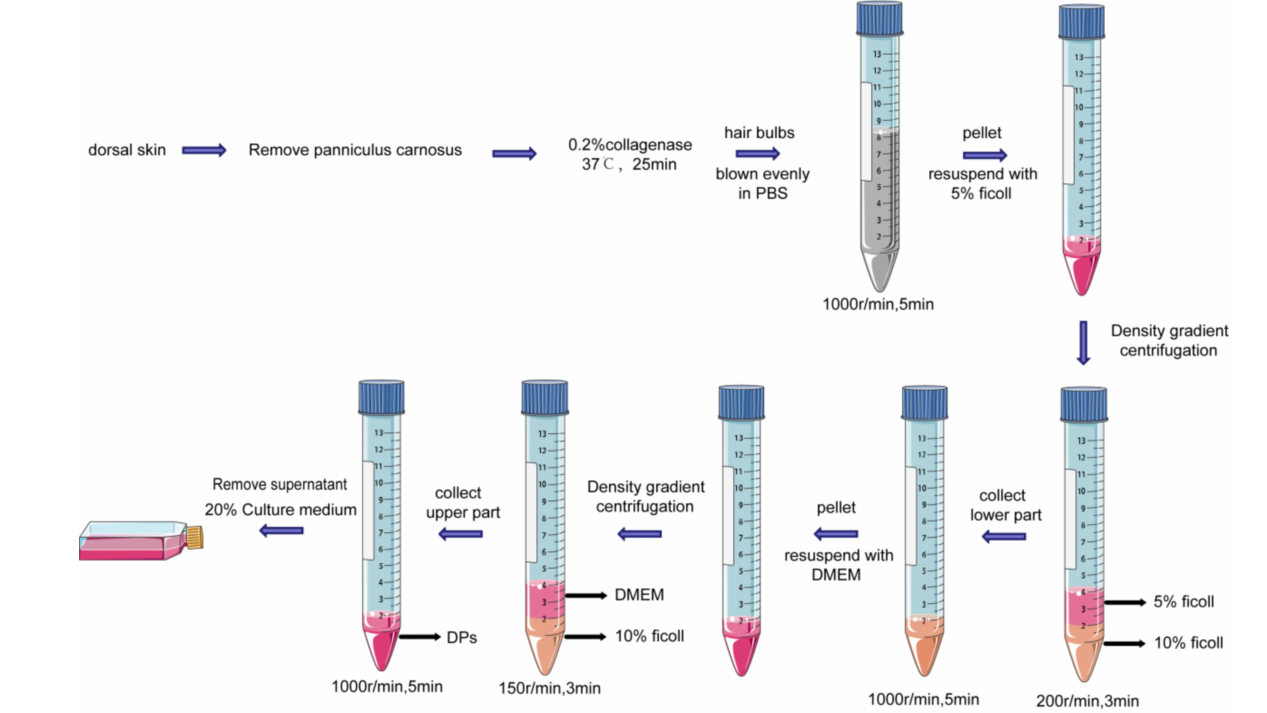


**Figure S2 Morphology, adhesion and cell migration of dermal papilla cells**

Whisker (A) and Pelage(B) follicles morphology observed under stereoscope, no blood sinus was found in pelage follicle, the number of pelage follicles were much more than whisker follicles. Hair shafts and hair bulb located in the same suspensions after enzyme digestion and scarping(C). After two-steps isolation, several Pelage DP spheres can be seen under microscope(D-F, Red arrow). Whisker DP spheres were observed under microscope(G, white arrow). Pelage DPCs started emigration in 24h(H, white arrow showed DPCs started emigrated out of the DP sphere) and proliferation, most of pelage DPCs finished emigration in 4 days(I, white arrow showed plenty of DPCs emigrated out of the DP sphere), while whisker DPCs started emigrate in 72h and finished in 7 days(J). Pelage DPCs(K) and whisker DPCs(L) showed similar cell morphology and aggregation growth pattern.


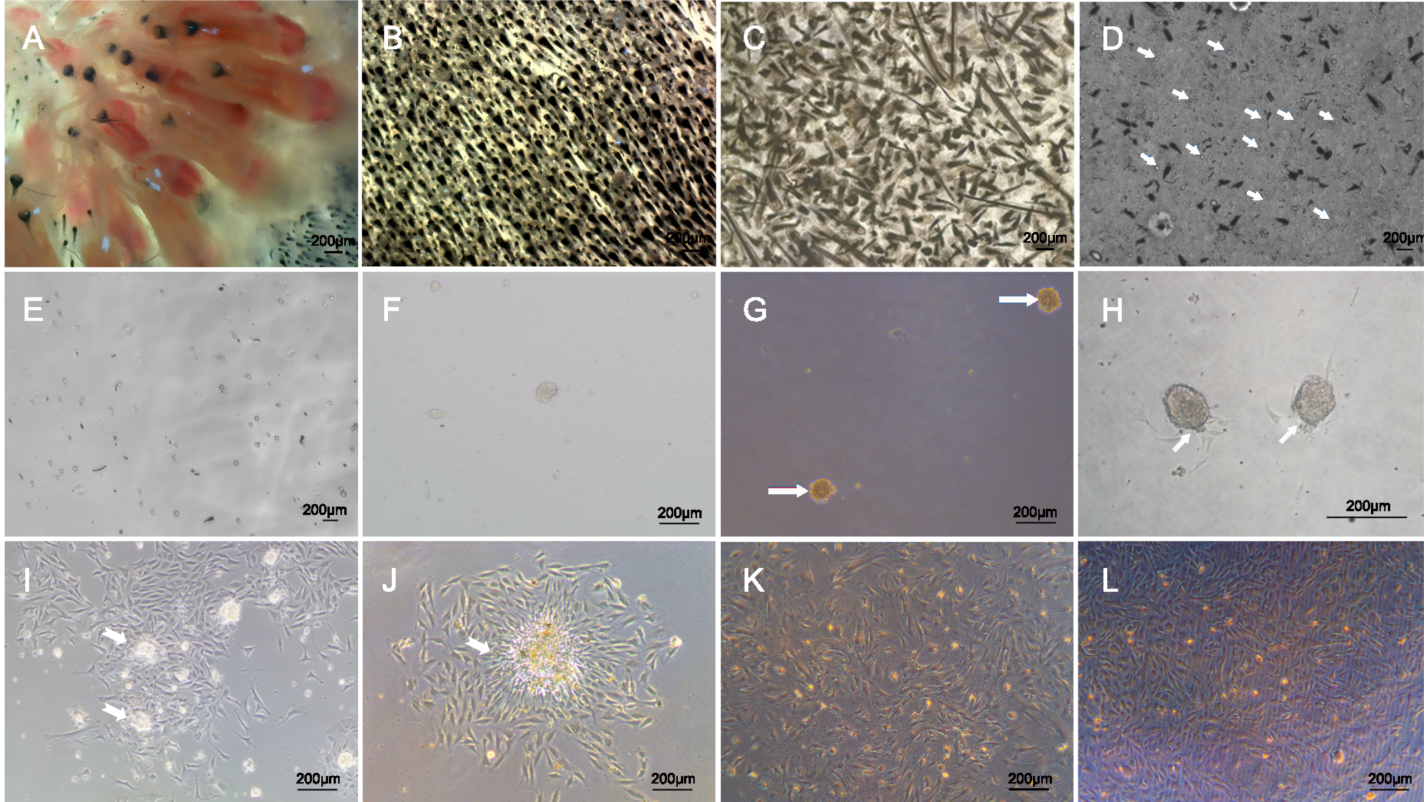


**Figure S3 Differential expression of Sox2 between whisker and pelage DPCs**

Sox2 expression was detected in primary DP sphere and cultured DPCs. Whisker DP showed high Sox2 expression both in DP sphere and DPCs, while Pelage DP showed almost no expression on Sox2 whether in primary DP sphere or cultured DPCs.


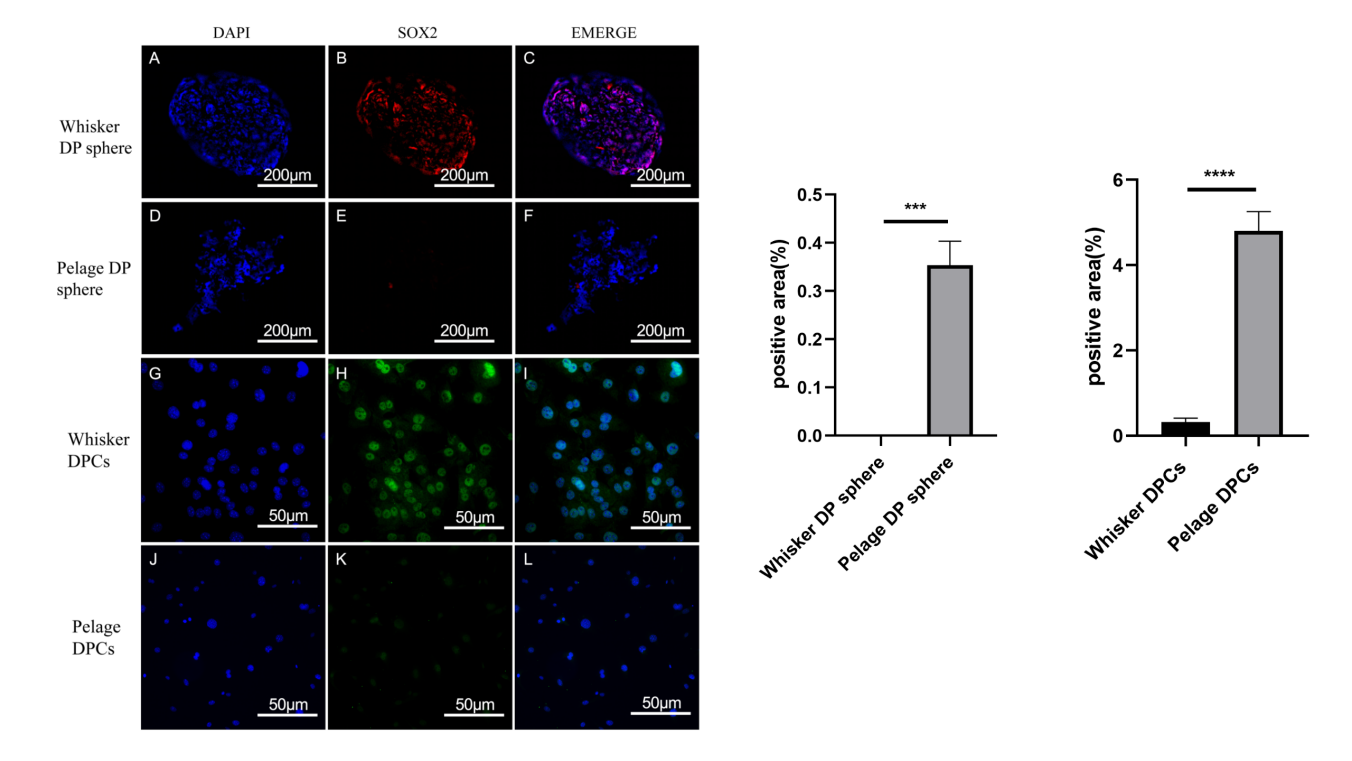


**Figure S4 Isolation and identification of Pelage HF-MSC and whisker HF-MSC exosomes.**

The morphology of exosomes was observed by electron microscope; Western Blotting verification specific markers CD9 and CD81 ; The detection particle size was between 30-100nm


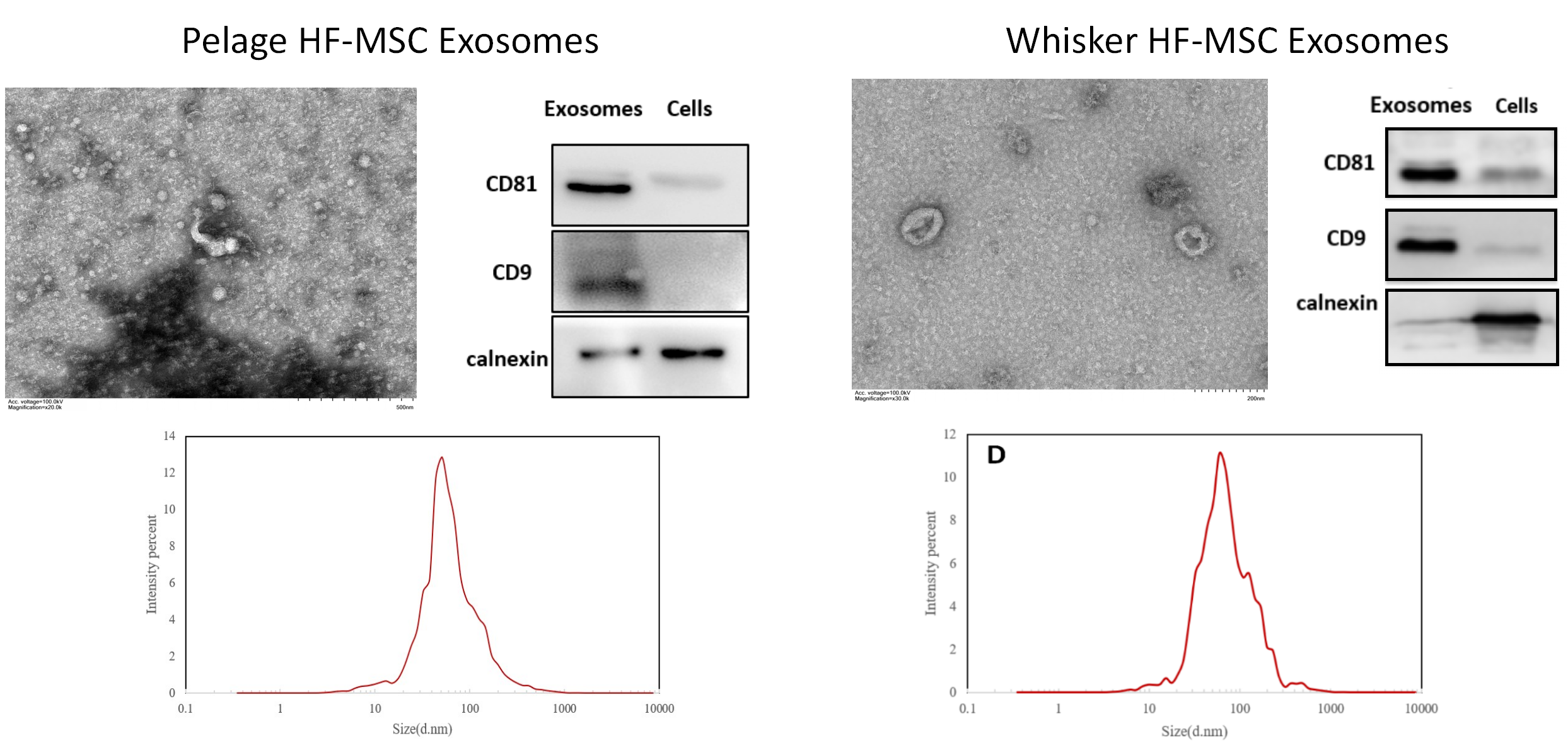


**Figure S5 Effect and distribution of Whisker HF-MSC treatment on dorsal hair growth.**

3-week-old C57BL/6 mice were divided into Pelage HF-MSC group and control group. Pelage HF-MSC group enter into anagen period earlier than control group, which was similar to Pelage HF-MSC group. Labeling whisker HF-MSC with DIO (green), showed that whisker HF-MSC located around the host hair follicle (White arrow).


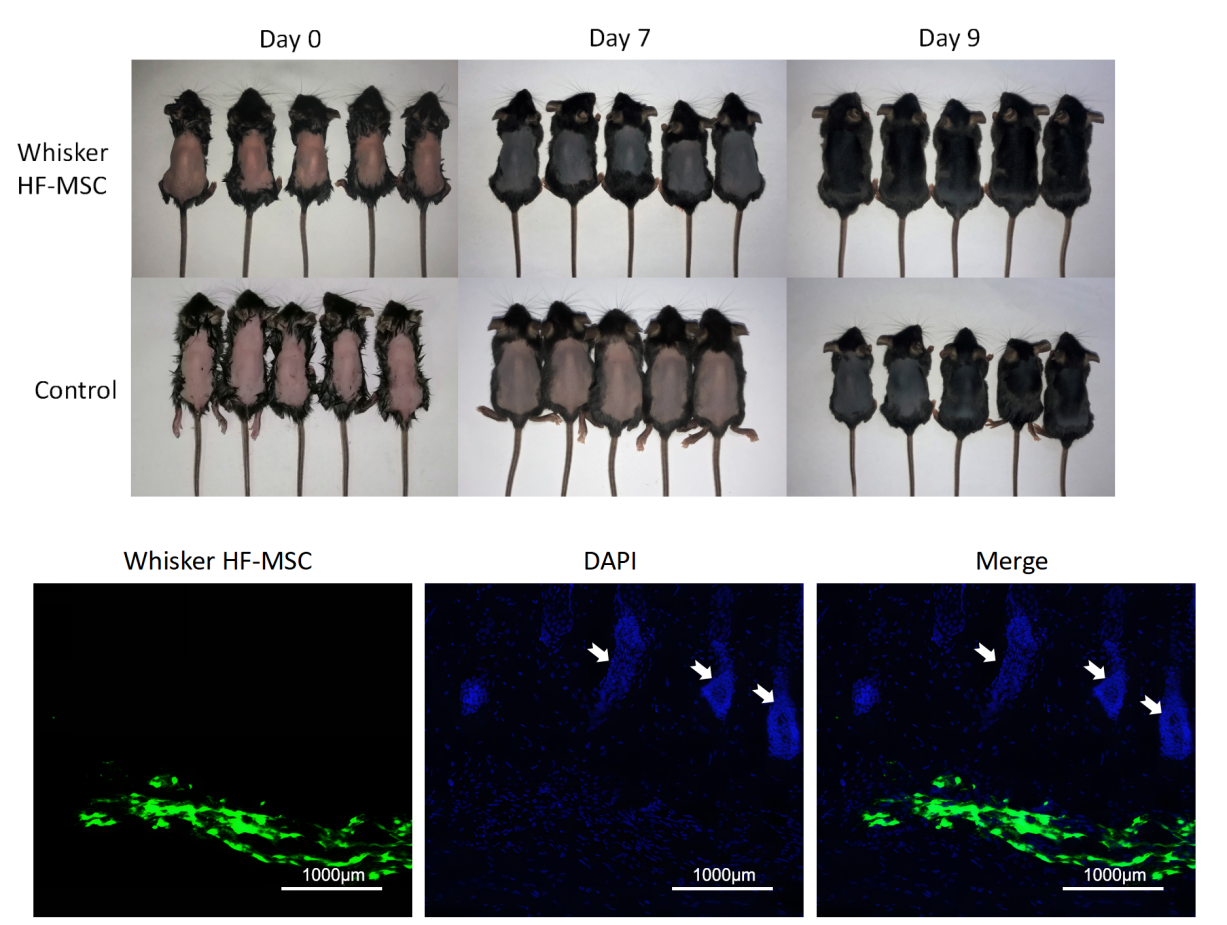


**Figure S6 Effect and distribution of whisker HF-MSC Exosomes treatment on dorsal hair growth.**

7-week-old C57BL/6 mice were divided into whisker HF-MSC exosomes group and control group(The same as the control group in Figure 6). Exosomes were injected every other day, a total of 3 times injection were performed. On day 8, whisker HF-MSC exosomes group enter into anagen period earlier than control group. Injected whisker HF-MSC exosomes were labeled with DIO (green) and located around host hair follicle (white arrow).


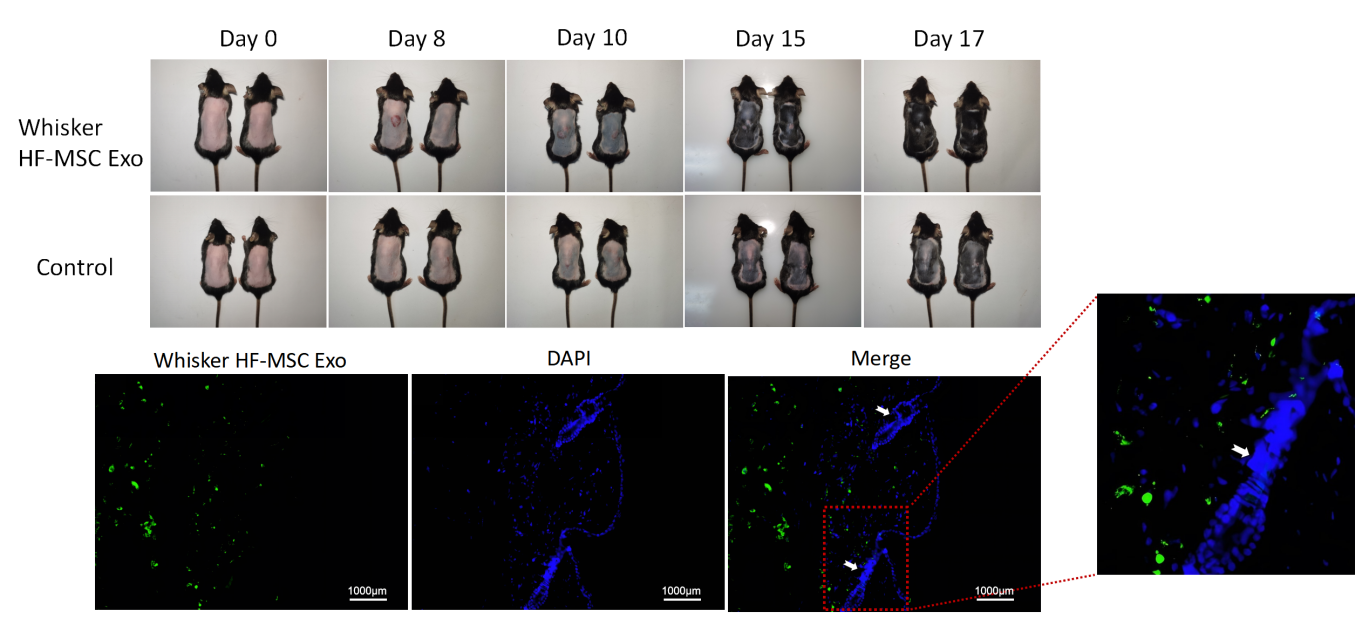


**Figure S7 Pictorial diagram to show the location of MSCs and exosomes injection protocol**

The cell/exosomes suspension was injected along the midline of the back of the mouse, and the amount of cell suspension was 300ul. HF-MSCs suspension injection performed once, Exosomes suspension injection performed.


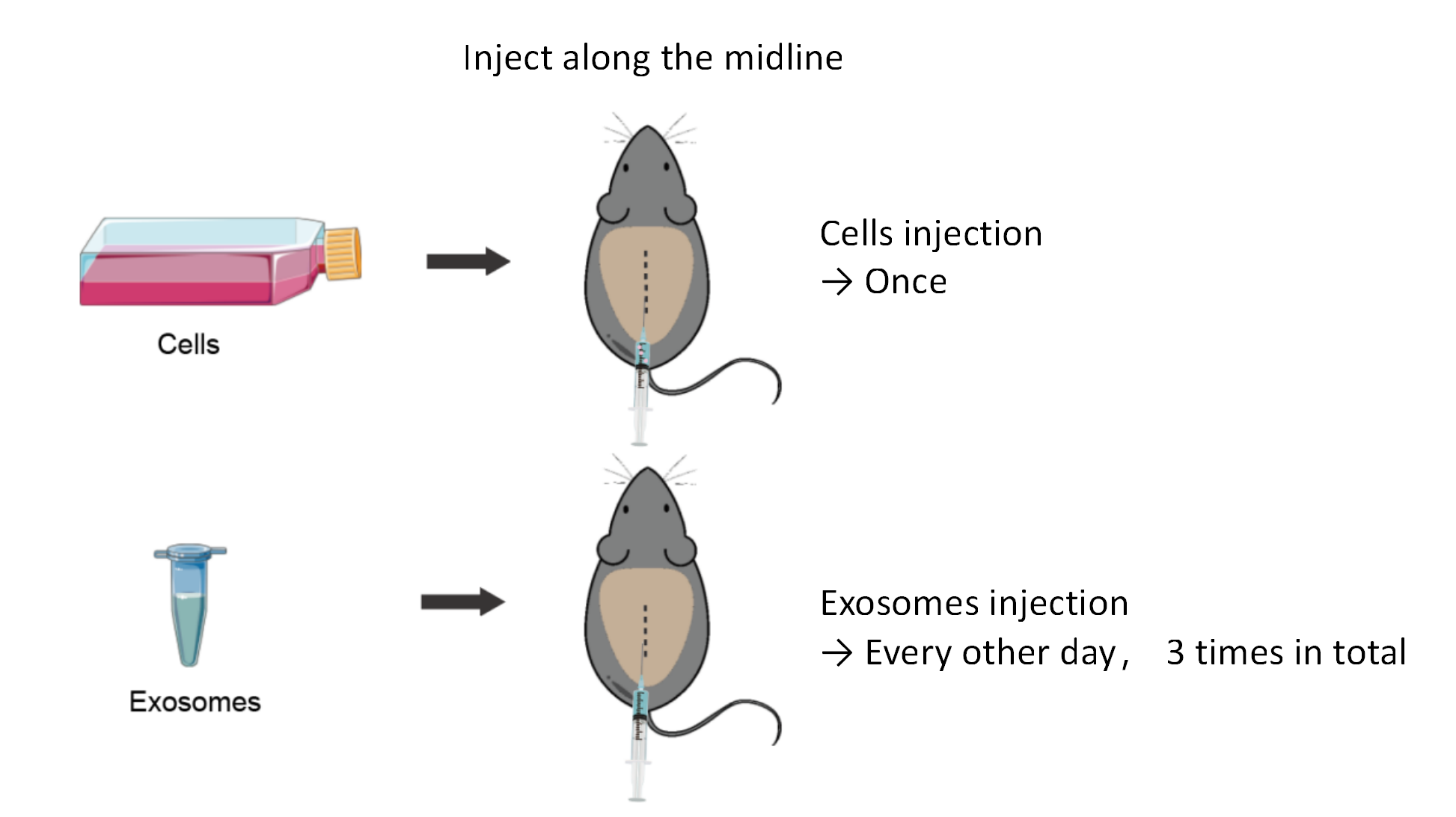

Supplement: Supplementary file 1 — Additional file 1: Fig. S1. Schematic diagram of Pelage DPCs isolation. Fig. S2. Morphology, adhesion and cell migration of dermal papilla cells. Whisker (A) and Pelage (B) follicles morphology observed under stereoscope, no blood sinus was found in pelage follicle, the number of pelage follicles was much more than whisker follicles. Hair shafts and hair bulb located in the same suspensions after enzyme digestion and scarping (C). After two-steps isolation, several Pelage DP spheres can be seen under microscope (D-F, Red arrow). Whisker DP spheres were observed under microscope (G, white arrow). Pelage DPCs started emigration in 24 h (H, white arrow showed DPCs started emigrated out of the DP sphere) and proliferation, most of pelage DPCs finished emigration in 4 days (I, white arrow showed plenty of DPCs emigrated out of the DP sphere), while whisker DPCs started emigrate in 72 h and finished in 7 days (J). Pelage DPCs (K) and whisker DPCs (L) showed similar cell morphology and aggregation growth pattern. Fig. S3. Differential expression of Sox2 between whisker and pelage DPCs. Sox2 expression was detected in primary DP sphere and cultured DPCs. Whisker DP showed high Sox2 expression both in DP sphere and DPCs, while Pelage DP showed almost no expression on Sox2 whether in primary DP sphere or cultured DPCs. Fig. S4. Isolation and identification of Pelage HF-MSC and whisker HF-MSC exosomes. The morphology of exosomes was observed by electron microscope; Western Blotting verification specific markers CD9 and CD81; The detection particle size was between 30 and 100 nm. Fig. S5. Effect and distribution of Whisker HF-MSC treatment on dorsal hair growth. 3-week-old C57BL/6 mice were divided into Pelage HF-MSC group and control group. Pelage HF-MSC group enter into anagen period earlier than control group, which was similar to Pelage HF-MSC group. Labeling whisker HF-MSC with DIO (green), showed that whisker HF-MSC located around the host hair follicle (White arrow). Fig [file 13287_2022_3051_MOESM1_ESM.docx]
